# The Composition and Primary Metabolic Potential of Microbial Communities Inhabiting the Surface Water in the Equatorial Eastern Indian Ocean

**DOI:** 10.3390/biology10030248

**Published:** 2021-03-22

**Authors:** Changling Ding, Chao Wu, Congcong Guo, Jiang Gui, Yuqiu Wei, Jun Sun

**Affiliations:** 1 College of Biotechnology, Tianjin University of Science and Technology, Tianjin 300457, China; docean@mail.tust.edu.cn; 2 Research Centre for Indian Ocean Ecosystem, Tianjin University of Science and Technology, Tianjin 300457, China; wuchao@tust.edu.cn (C.W.); smart_guo@mail.sdu.edu.cn (C.G.); gui18825008@mail.tust.edu.cn (J.G.); 201720544@mail.sdu.edu.cn (Y.W.); 3 Institute of Marine Science and Technology, Shandong University, Qingdao 266237, China; 4 College of Marine Science and Technology, China University of Geosciences (Wuhan), Wuhan 430074, China

**Keywords:** eastern Indian Ocean, microbial community, *Prochlorococcus*, microbial metabolism, metagenome

## Abstract

**Simple Summary:**

Marine microbes are regarded as the most diverse organisms in the biosphere and drive biogeochemical cycles through their metabolism. It is essential to understand the structure and metabolic function of microbial communities. The Indian Ocean is the third largest ocean in the world, and it possesses unique hydrographical properties. So far, assessments of microbial diversity and metabolism need to be improved in the Indian Ocean. Therefore, we carried out a series of investigations in the equatorial eastern Indian Ocean in order to clarify the local microbial communities and detect the genetic potential for microbial functions. The obtained results suggested Cyanobacteria was the dominant microbial group, and predicted the Calvin cycle and the assimilatory nitrate and nitrite reduction played important role in the pathway of carbon fixation and nitrogen metabolism respectively. This study provides insights into microbial community structures as well as the metabolic potential that may be active in the local environment, and lays the groundwork for understanding the roles of microbes in energy and resource cycling in this habitat.

**Abstract:**

Currently, there is scant information about the biodiversity and functional diversity of microbes in the eastern Indian Ocean (EIO). Here, we used a combination of high-throughput sequencing of 16S rRNA genes and a metagenomic approach to investigate the microbial population structure and its metabolic function in the equatorial EIO. Our results show that Cyanobacterial *Prochlorococcus* made up the majority of the population. Interestingly, there were fewer contributions from clades SAR11 (Alphaproteobacteria) and SAR86 (Gammaproteobacteria) to microbial communities than contributions from *Prochlorococcus*. Based on functional gene analysis, functional genes *rbcL*, *narB*, and *nasA* were relatively abundant among the relevant genes. The abundance of *Prochlorococcus* implies its typically ecological adaptation in the local ecosystem. The microbial metabolic potential shows that in addition to the main carbon fixation pathway Calvin cycle, the rTCA cycle and the 3-HP/4-HB cycle have potential alternative carbon fixation contributions to local ecosystems. For the nitrogen cycle, the assimilatory nitrate and nitrite reduction pathway is potentially the crucial form of nitrogen utilization; unexpectedly, nitrogen fixation activity was relatively weak. This study extends our knowledge of the roles of microbes in energy and resource cycling in the EIO and provides a foundation for revealing profound biogeochemical processes driven by the microbial community in the ocean.

## 1. Introduction

Marine microbes, as the most diverse organisms, have unparalleled importance for marine ecosystem, they play vital roles in the biogeochemical pathways in the ocean, including the modulation of atmospheric CO_2_ and the efficiency of nutrient utilization [1,2,3]. At present, much finer scales of microbial community have been advanced [4,5], with the aid of the development of new technical and modeling approaches [6,7,8,9,10,11]. Modern researches provide more comprehensive interpretations of marine microbial populations and interactions with complex environmental properties, as well as the ecological and biogeochemical importance of the diverse marine microbial populations [4,12,13,14,15]. Recently, comprehensive studies on marine microbes have been performed by several world’s projects [10,15,16,17,18,19]. For instance, the Sorcerer II Global Ocean Sampling (GOS) project developed new comparative genomic and assembly methods, and facilitated the study of surface aquatic microbial communities from the northwest Atlantic to the eastern tropical Pacific [10,18]. The genomic content of prevalent microbiota was captured across major oceanic regions in the Tara Oceans project, and the vast majority of genes uncovered in the Tara Oceans samples had been previously unidentified, with a particularly high fractions of novel genes [4,12]. Many studies on marine microbes have been carried out on a global scale, however related studies are still rudimentary in certain regional oceans.

As one of the three major oceans, the Indian Ocean is relatively poorly investigated due to dynamically complex and highly variable surroundings.

The most striking feature of the Indian Ocean is the seasonal reversal of the monsoon winds [20]. The typical physical processes in the tropical Indian Ocean, such as Wyrtki jets, Madden–Julian oscillation, and the Indian Ocean Dipole, stimulate dynamic and thermodynamic responses [21,22,23,24]. Such dynamic ocean conditions lead to altered patterns of biodiversity and ecosystem functions [25]. Previously, researchers characterized the abundance, diversity, and functions of bacteria in the Indian Ocean by isolation of the cultivable species [26,27]. Recently, studies on environmental microbes were performed from water columns and sediments [28,29,30,31,32,33,34]. Wang et al. (2016) and Qian et al. (2018) provided an extensive depiction of bacteria in their environmental context [28,31]. Shiozaki et al. (2014) and Wu et al. (2019) observed that heterotrophic bacteria were major nitrogen fixers in the euphotic zone [30,33]. The Tara Oceans project revealed the distinct environmental adaptations of microbial communities in the northern Indian Ocean [4]. Existing scientific discoveries have led to major advances in understanding the interaction of biogeochemical processes and the specific microbial populations responsible for these processes. 

In fact, microbial community in the equatorial Eastern Indian Ocean (EIO) is relatively sparsely studied compared with the other oceans. The picture of the metabolic capabilities and microbial functional diversity is not yet fully resolved especially in the equatorial EIO. Here, samples were collected along the equator during the presouthwest monsoon of 2019, aiming to explore the microbial identity and the vital roles that microbes may play in the local ocean. Besides 16S rDNA analysis, a metagenomic method which is well suited expand knowledge of ecology potential of the natural marine microbes [35,36], was adopted to characterize the potential ecological functions of microbial communities in the equatorial EIO. The main objectives were attempt to, (1) describe the composition and diversity of natural microbes, (2) sketch the potentially metabolic profiles of microbial communities, (3) predict potential functions of these microbes in energy and resource cycling. This study provided insight into the contribution of microbial communities to biogeochemical cycling in the EIO. 

## 2. Materials and Methods

### 2.1. Station Location, Sample Collection, and Environmental Parameter Measurements

**Figure 1:**
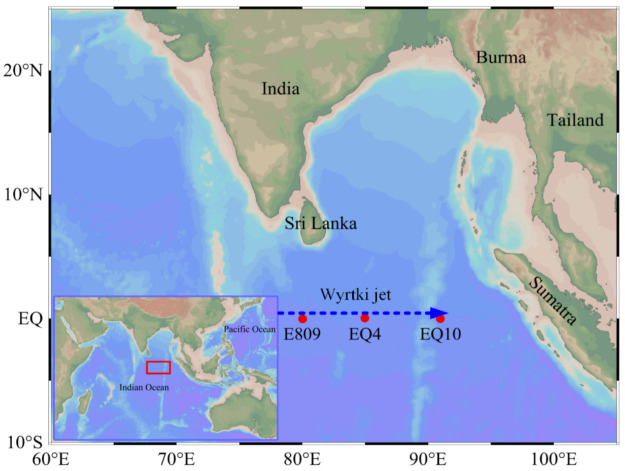
Map of sampling stations (red dots) and Wyrtki jet (blue dotted arrow) along the equatorial EIO.

The multidisciplinary cruise was carried out in the EIO onboard R/V “Shiyan 3” from 20 March to 6 June 2019. Three stations (defined as E809, EQ4, and EQ10) were selected for the surface water sampling along the equator (Figure 1). Water samples were collected by using 5 L Teflon-coated Go-Flo bottles (General Oceanics, Miami, FL, USA), which were attached to a rosette multisampler, on which a conductivity–temperature–depth (CTD) system was installed (Seabird SBE 911Plus, Sea-Bird Electronics, Bellevue, WA, USA). 

Surface water was collected by using 10% hydrochloric acid (HCl)-rinsed polyethylene (PE) bucket at all the stations. Samples for pyrosequencing analyses were achieved by filtering 60 L of seawater through 0.22 μm filters (GTTP, 142 mm in diameter, Merck Millipore, Germany) under vacuum pressure of less than 13 kPa. Filters were placed into 5 mL tubes with sterilized forceps and stored in liquid nitrogen immediately. Then, these filters were transported to the laboratory on dry ice and stored at −80 °C for downstream analysis. For sampling for nutrient analysis, collecting seawaters were transferred into 100 mL HCl-rinsed bottles and stored at −20 °C immediately until analyzed in the lab. For chlorophyll *a* (Chl *a*) analysis, 1 L samples from each station were vacuum-filtered (<10 mm Hg) through 25 mm Waterman GF/F filters and stored in the dark at −20 °C until analyzed in the laboratory.

Temperature and salinity were measured and recorded vertically by a CTD profiler in situ. The Chl *a* samples were analyzed immediately in the laboratory using the fluorescence method followed by Parsons et al. (1984) [37]. The Chl *a* filters were extracted with 90% acetone and extracts were then refrigerated at 4 °C for 24 h, after which Chl *a* concentrations were measured using a Turner Designs Thilogy^TM^ laboratory fluorometer (Turner Designs, San Jose, CA, USA). Nutrient concentrations, including nitrate (NO_3_^−^), nitrite (NO_2_^−^), phosphate (PO_4_^3−^), silicate (SiO_3_^2−^), and ammonium (NH_4_^+^), were measured using a Technicon AA3 Auto-Analyzer (SEAL Analytical, Norderstedt, Germany) based on classical colorimetric methods. Concentrations of ${NO}_{3}^{-}$ and ${NO}_{2}^{-}$ were measured using copper-cadmium column reduction methods, whereas concentrations of PO_4_^3−^, SiO_3_^2−^, and NH_4_^+^ were measured by the phosphomolybdate complex method, the silicomolybdate complex method, and the indophenol blue method, respectively [38].

### 2.2. DNA Extraction and Sequencing of 16S rDNA

The genomic DNA was extracted from three selected surface water samples using DNeasy PowerWater^®^ Kit (Qiagen, Hilden, Germany) following the manufacturer’s protocols. The V3-V4 region of the bacteria 16S ribosomal RNA genes were amplified by PCR using primers 338F 5′-barcode- ACTCCTACGGGAGGCAGCAG)-3′ and 806R 5′- GGACTACHVGGGTWTCTAAT -3′ [39], where barcode is an eight-base sequence unique to each sample. DNA Amplification was performed with the ABI GeneAmp^®^ 9700 thermocycler (Applied Biosystems, Foster City, CA, USA). Then, purified amplicons were pooled in equimolar and paired-end sequenced (2 × 250) on an Illumina MiSeq platform according to the standard protocols. The taxonomy of each 16S rDNA gene sequence was analyzed by RDP Classifier (http://rdp.cme.msu.edu/, RDP Taxanomy 18, accessed on 14 August 2020) against the Silva (SSU115) 16S rDNA database using a confidence threshold of 70%. The more detailed information of research method were shown in the Supplementary File S1. The raw reads were deposited into the NCBI Sequence Read Archive (SRA) database (BioProject: PRJNA685401, SUB8740811). All libraries were constructed and sequenced via paired-end chemistry (PE300) on an Illumina Miseq platform (Illumina, San Diego, CA, USA) at ZhongKe BlueOcean (TianJin) Technology Co. Ltd., Tianjin, China.

### 2.3. Metagenomics Sequencing, and Genome Assembly

DNA for metagenomics was fragmented to an average size of about 300 bp using Covaris M220 (Gene Company Limited, China) for paired-end library construction. The paired-end library was prepared by using the TruSeq^TM^ DNA Sample Prep Kit (Illumina, San Diego, CA, USA). Adapters containing the full complement of sequencing primer hybridization sites were ligated to the Blunt-end fragments. Paired-end sequencing was performed on the Illumina NovaSeq platform (Illumina Inc., San Diego, CA, USA) according to the manufacturer’s instructions (https://support.illumina.com.cn/custom-protocol-selector.html, accessed on 14 August 2018). Contigs obtained after quality control were used for further gene prediction and annotation. The detailed laboratory procedures were shown in the Supplementary File S1. Genome binning was performed with contigs which were spliced and aligned from the original short-read sequence by using the MetaWRAP software [40]. The quality of the assembled bins was evaluated by CheckM method, and high-quality ones were chosen to conduct preliminary species identification, completeness, and pollution assessment [41]. All the raw metagenomics datasets have been deposited into the NCBI Sequence Read Achieve database (BioProject: PRJNA685401, SUB8742649).

### 2.4. Gene Prediction, Taxonomy, and Functional Annotation

Open reading frames (ORFs) from each metagenome sample were predicted using MetaGene (http://www.bioconductor.org/packages/release/bioc/html/metagene.html, Bioconductor version 3.12, accessed on 28 October 2020) [42]. The predicted ORFs with lengths being or over 100 bp were retrieved and translated to amino acid sequences using the NCBI translation table (http://www.ncbi.nlm.nih.gov/Taxonomy/taxonomyhome.html/index.cgi?chapter=tgencodes#SG1, accessed on 7 January 2019). All sequences from gene sets with a 95% sequence identity (90% coverage) were clustered as the nonredundant gene catalog by CD-HIT (http://www.bioinformatics.org/cd-hit/, accessed on 1 September 2009) [43]. Reads after quality control were mapped to the representative genes with 95% identity using SOAPaligner (https://github.com/aquaskyline/SOAPdenovo2, accessed on 14 august 2015), and gene abundance in each sample was evaluated [44]. BLASTP (Version 2.2.28+, http://blast.ncbi.nlm.nih.gov/Blast.cgi, accessed on 23 February 2021) was employed for taxonomic annotations by aligning nonredundant gene catalogs against the NCBI NR database with an e-value cutoff of 1 × 10^−5^ [45]. Cluster of orthologous groups of proteins (COG) for the ORF annotation was performed using BLASTP against the eggNOG database (v4.5) with an e-value cutoff of 1 × 10^−5^ [46,47]. The KEGG pathway annotation was conducted using BLASTP search (Version 2.2.28+) against the Kyoto Encyclopedia of Genes and Genomes database (https://www.genome.jp/kegg/, accessed on 1 January 2021) with an e-value cutoff of 1 × 10^−5^ [48].

### 2.5. Data Analysis

Sequences recovered from 16S rDNA libraries were blasted in GenBank using BLAST (https://blast.ncbi.nlm.nih.gov/Blast.cgi, accessed on 23 February 2021). The representative sequences and alignment sequences were aligned with Clustal W in MEGA X [49], and a phylogenetic neighbor-joining tree was subsequently constructed using the maximum likelihood method, based on the OTU data. Bootstrap values were determined by resampling 1000 times. The constructed tree was further edited by Interactive Tree of Life (iTOL), an online tool for managing phylogenetic trees [50]. The circus and hot map were drawn in the cloud platform (https://www.omicshare.com/tools/, accessed on 3 November 2019). Bar charts in this study were created with OriginPro 2020.

## 3. Results

### 3.1. Environmental Parameter, Sequencing Statistics and Diversity Estimates

During this study, environmental parameters were comparable among the three sites (Table 1). By 16S rDNA sequencing, a total of 160,742 DNA sequences were included in our study after quality control. Based on 97% similarity, a total of representative 447 OTUs were obtained from the cluster analysis in non-repeating sequences (excluding single sequences). From the combined alpha diversity indices, Stn. E80-9 was found to have the highest diversity indices, while the lowest indices occurred in Stn. EQ04. The rarefaction curves of diversity indices are performed in the Supplementary Figure S1. From metagenomic analysis, samples targeting microbes were paired-end sequenced to generate a total of more than 12.3 Gb per sample. We totally obtained 759408 catalog genes. A total of 258,782,775, 299,252,706, and 228,477,045 bp protein-coding nucleotide sequences were predicted in Stn. E80-9, EQ04, and EQ10, respectively. Specifically, a total of 759,408 catalog genes were included in our study, and the detailed information for each sample is listed in Table 2.

### 3.2. Composition of Microbial Community

**Figure 2:**
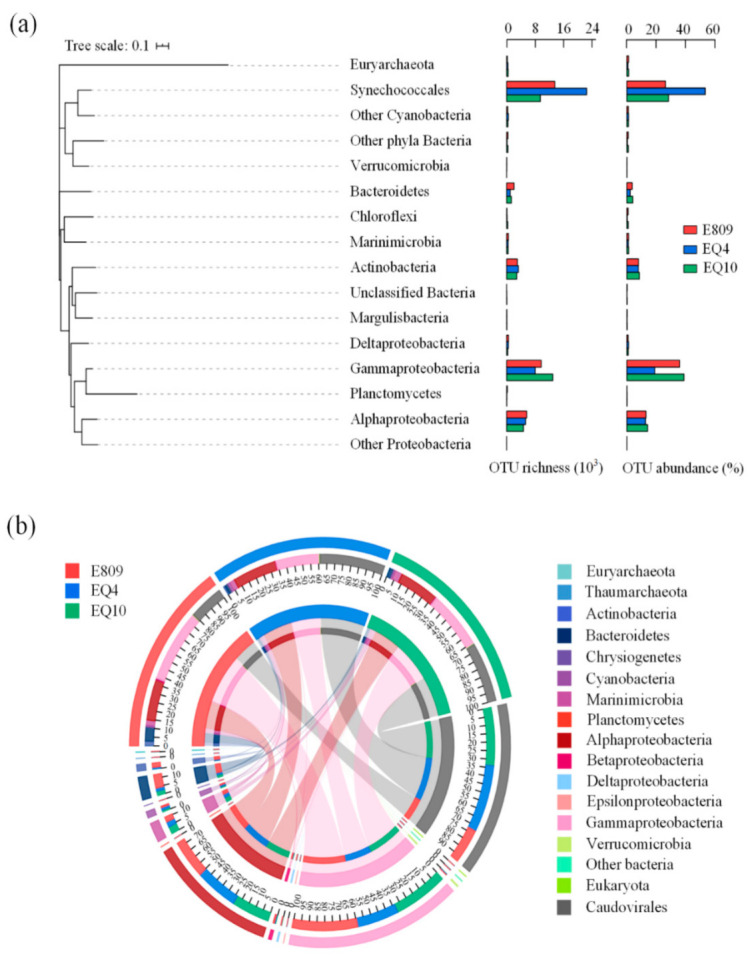
Taxonomic analysis and the number of detected taxa. A phylum-level (class-level for Proteobacteria) breakdown of relative abundances is shown for all samples, along with the number of detected taxa at the OTU level (**a**), bar graphs represent richness and abundance (%) of OTUs from three samples. The circos map shows different microbes contributes to the community based on microbe metagenome data (**b**), the outer semicircle above represents three samples, and inner semicircle marked scales represents the relative proportions of different microbes in the community.

**Figure 3:**
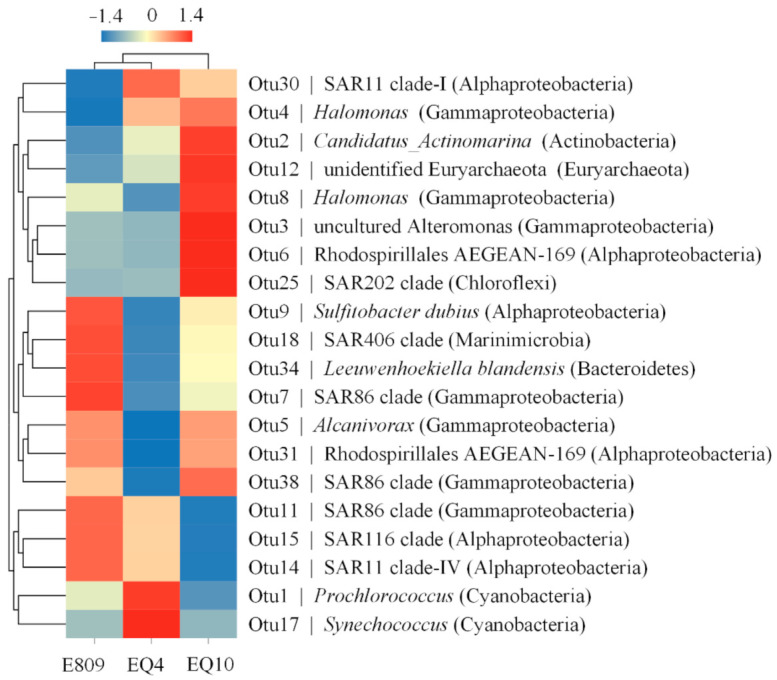
Heat map based on top 20 sequence abundance of genera (OTUs). The color density represents the normalized value of each taxon abundance (%) in the three samples (−1.4 means the minimum value, 1.4 means the maximum value).

From 16S rDNA data, microbial communities were mainly composed of Proteobacteria, Cyanobacteria, Actinobacteria, Bacteroidetes, Marinimicrobia, and archaeal Euryarchaeota at the phylum level (Figure 2a). Bacterial playlotypes dominated the community with relatively small proportions of Archaea at the targeting sampling stations. Proteobacteria and Cyanobacteria were two main groups in the sampled areas both in terms of relative abundance and taxonomic richness. *Synechococcales*, Gammaproteobacteria, and Alphaproteobacteria were preponderant based on 16S rDNA analysis. The sum of Proteobacteria and Cyanobacteria relative abundance was approximately 84% on average, and Gammaproteobacteria constituted a similar amount as *Prochlorococcus* (Figure 2a). Besides, among top 20 sequence abundance of OTUs, OTU 1 annotated to *Prochlorococcus* contributed to 36.42%, 51.57%, and 27.28% in Stn E809, EQ4, and EQ10 respectively. OTU 17 annotated to *Synechococcales* was also relatively abundant in the Stn EQ4 (Figure 3), which contributed 1.01%, 1.56% and 0.98% in Stn E809, EQ4, and EQ10 respectively. OTU 3 and OTU 8 annotated to *Alteromonas* and *Halomonas* (Gammaproteobacteria) respectively were relatively abundant in the Stn EQ10 (contributed 11.29% and 2.54% respectively). OTU 9 annotated to *Sulfitobacter* (Alphaproteobacteria), as well as OTU 7 annotated to SAR86 (Gammaproteobacteria) were relatively abundant in the Stn E809 (contributed 1.58% and 1.51% respectively).

Further, the abundant Proteobacteria in the microbial community based on metagenomics data was approximately in line with that based on 16S rDNA data. Among the metagenomics data, genes annotated to Gammaproteobacteria (40.88%, 26.01%, and 33.50% at Stn E809, EQ4, and EQ10, respectively) and Alphaproteobacteria (22.82%, 26.26%, and 23.66% at Stn E809, EQ4, and EQ10, respectively) were dominant (Figure 2b). The difference between two results was that superiority of Cyanobacteria was obscure in the metagenomics data. Moreover, a total of five high-quality genomes (completeness > 98% and contamination < 1%) were reconstructed from the studied samples after assembly and binning (Table 3). The results show that relatively high-completeness bin.59, bin.46, and bin.15 belonged to Gammaproteobacteria. These five reconstructed genomes have been deposited into the NCBI database (SUB9213355).

### 3.3. The Snapshot of Microbial Community Function

**Figure 4:**
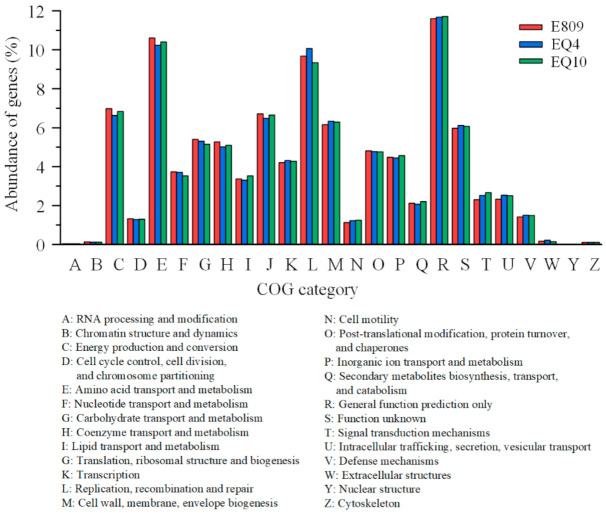
The predicted genes in the metagenome were matched to the functional categories in the COG database.

**Figure 5:**
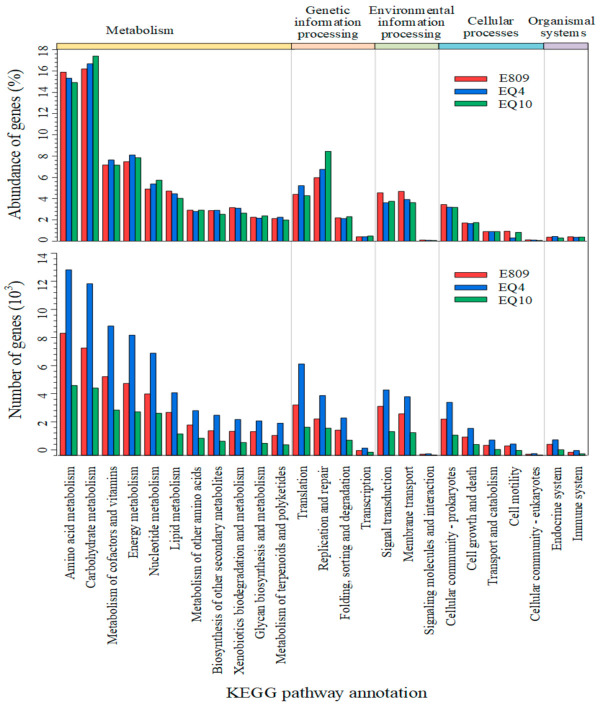
The predicted genes in the metagenome were matched to the functional categories in the KEGG database.

The predicted genes in the metagenomes were functionally annotated to orthologous groups in the eggNOG and KEGG databases. No obvious difference was observed in the relative abundance of the annotated functional genes among the three samples. In total, 73.06% and 33.35% of the genes could be annotated to the COG database and KEGG ortholog group (KO), respectively. The majority of functional compositions based on COG annotation were classified into general function prediction only (R); amino acid transport and metabolism (E); replication, recombination, and repair (L); and energy production and conversion (C), among which the relative abundances of functional genes annotated to R, E, and L levels were above 11.60%, 10.24%, and 9.34%, respectively (Figure 4). Functional profiling using KEGG annotation revealed that the highest relative abundance of genes contributed to the metabolism functional categories. Functional genes classified into carbohydrate metabolism, amino acid metabolism, energy metabolism, and metabolism of cofactors and vitamins were dominant among the subcategory level. Further, functional genes belonging to the level of replication and repair had a relatively large proportion, among which carbohydrate metabolism was the most major function of the microbial community, the relative abundances of which genes were 16.19%, 16.68%, and 17.41% at sites E809, EQ4, and EQ10, respectively. For amino acid metabolism, one of the major microbial functions, the relative abundances of genes were also apparently higher than other functions, the values of which were 15.32%, 14.92%, and 15.89% at sites E809, EQ4, and EQ10, respectively (Figure 5).

### 3.4. Functional Genes and Metabolic Pathways Related to CO_2_ Fixation and Nitrogen Metabolism

**Figure 6:**
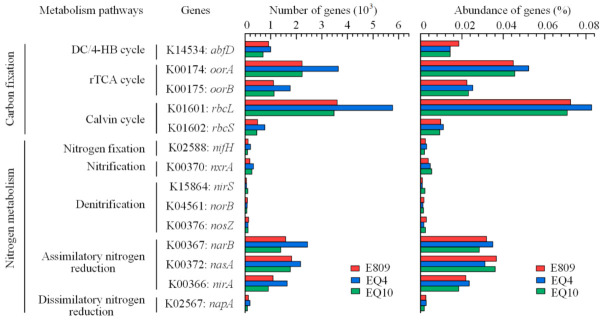
The relative abundances of genes encoding key enzymes of carbon fixation and nitrogen metabolism pathways.

Crucial metabolic pathways related to CO_2_ fixation and nitrogen cycle processes were analyzed by selecting key marker genes based on the KEGG database (Figure 6). The key enzyme genes of the CO_2_ fixation pathway, such as *abfD*, *oorA*, *oorB*, *rbcL*, and *rbcS*, were annotated in three metagenomes. The relative abundance and richness of *rbcL* and *rbcS* genes encoding ribulose bisphosphate carboxylase (EC:4.1.1.39) in the CO_2_ fixation pathway Calvin cycle were the highest among these functional genes, followed by the *oorA* and *oorB* genes encoding 2-oxoglutarate/2-oxoacid ferredoxin oxidoreductase (EC:1.2.7.3 1.2.7.11) in the reductive tricarboxylic acid cycle (rTCA cycle). The *abfD* gene encoding 4-hydroxybutyryl-CoA dehydratase/vinylacetyl-CoA-Delta-isomerase in the dicarboxylate/4-Hydroxybutyrate cycle (DC/4-HB cycle) was also relatively abundant, although the richness within this functional gene was smaller. These results show that the Calvin cycle and the rTCA cycle were the two main CO_2_ fixation pathways potentially in the study area. For nitrogen metabolism, functional genes related to five nitrogen metabolism pathways were annotated from metagenome analysis, among which the abundances of functional genes (*narB*, *nasA*, and *nirA*) controlling assimilatory nitrogen reduction were much higher than other nitrogen metabolism pathways, suggesting that assimilatory nitrogen reduction was the important process in nitrogen metabolism. By contrast, the proportions of key genes participating in nitrification (*nxrA*), nitrogen fixation (*nifH*), and dissimilatory nitrogen reduction (*napA*) were relatively low but exceeded the proportions of functional genes (*nirS*, *norB*, and *nosZ*) involved in denitrification.

## 4. Discussion

### 4.1. Characterization of Microbial Community Structure

The roles of these microbes were emphasized in energy and resource cycling in the eastern Indian Ocean recently. Wang et al. (2016) observed that Bacteroidetes, Proteobacteria (mainly Alpha and Gamma), Actinobacteria, Cyanobacteria, and Planctomycetes dominated the microbial communities generally, and detailed data suggest that Cyanobacteria and Actinobacteria are more predominant in surface water [31]. The application of 16S rDNA gene analysis and metagenomic strategies in this study provides more information about the genetic diversity of microbes that dominate in the EIO. We observed that phyla Proteobacteria (mainly Gammaproteobacteria and Alphaproteobacteria) and Cyanobacteria were predominant in the equatorial EIO. Among Proteobacteria, Gammaproteobacteria *Halomonas* and *Alteromonas* contributed a relatively high proportion to the microbial community. In other oceans, Proteobacteria dominate ocean surface microbial communities, and the taxonomically rich Cyanobacteria, Deferribacteres, and Thaumarchaeota are also abundant, although the taxonomic richness within these phyla is smaller [4]. Previous studies suggested that prokaryotic community composition was primed by the formation and the horizontal transport of water masses, and the success of microbial populations were highly governed by specialized adaptations and interactions in the local oligotrophic ocean [51,52]. In the equatorial EIO, typical intraseasonal or interannual physical processes influenced the occurrence of distinctive microbial community composition. Unexpectedly, the clades SAR11 (Alphaproteobacteria) and SAR86 (Gammaproteobacteria) only accounted for a small proportion in this study, even though the clades SAR11 (Alphaproteobacteria) and SAR86 (Gammaproteobacteria) are found throughout the oceans and reach their largest numbers in stratified and oligotrophic gyres [53,54].

The unicellular cyanobacterial Oxyphotobacteria are the numerically dominant photosynthetic organisms throughout the tropical and subtropical oceans [55]. Previously, it was estimated that *Prochlorococcus* and *Synechococcus* were on average most abundant in warm oligotrophic waters, especially the Indian and western Pacific Ocean subtropical gyres using the parametric models [56]. We detected that the relative contribution of *Prochlorococcus* even reached half of the microbial community, whereas the relative contribution of *Synechococcus* to the microbial community was very small, indicating that *Prochlorococcus* were actively replicating in the survey area. Similarly, Díez et al. (2016) sequencing results obtained from Indian Ocean identified cyanobacteria accounted for up to 15% of annotated reads, with the genera *Prochlorococcus* and *Synechococcus* comprising 90% of the cyanobacterial reads [35]. Further, the sequencing abundance pattern of *Prochlorococcus* in this study was in line with the flow cytometry cell abundance data that *Prochlorococcus* numerically dominated the picophytoplankton assemblage [57]. There is evidence that the comparatively stable distribution and interannual abundance of *Prochlorococcus* play a role as a very good biological indicator of oligotrophic water masses [58,59,60,61]. Choi et al. (2012) identified that *Prochlorococcus* dominate the open ocean stations affected by the North Equatorial Current of the Pacific Ocean, indicating that water masses seem to be important in determining biodiversity [62]. In comparison, we can speculate that currents in the equatorial EIO potentially impacted the distribution of photosynthetic Cyanobacteria, representing *Prochlorococcus* to a certain extent.

### 4.2. Potential Function and Metabolism Properties of Microbes

The microbial community is very important for the way in which ecosystems function. It has been confirmed that microbes are closely relevant in carbon and nutrient cycling in the ocean [63,64,65]. We sought to explore the functions and metabolic potential of microbial communities in the surface equatorial EIO. Based on metagenomic data, we predicted that the most annotated genes belonged to the metabolism catalog, followed by genetic information processing. To understand the ecological importance of microbial communities, carbon fixation and nitrogen cycling were considered from the aspect of their functional genes. Our results provided a clue that the Calvin cycle is potentially the most significant carbon fixation pathway in the EIO. This prevailing fixation pathway that occurs in the photic zone was confirmed as the conserve energy strategy [66]. The significance of this cycle has been highlighted in *Prochlorococcus* on the account of the most highly expressed functional genes among in their genomes [67]. Recently, Pujari et al. (2019) revealed that, in the *rbcL* gene library, *rbcL* genes from Cyanobacteria *Prochlorococcus* are predominant near the equator in the EIO [68]. Associated with the community composition of this study, we speculate that dominant cyanobacteria, especially *Prochlorococcus*, potentially play an important role in local primary production by using the Calvin cycle for carbon fixation. In addition, identification of functional genes related to the rTCA cycle and the 3-HP/4-HB cycle in this study indicate their potential contributions to local ecosystems as alternative carbon fixation pathways.

With respect to nitrogen cycling, relevant genes of assimilatory nitrogen reduction pathways were enriched in the surface water; in contrast, functional genes of nitrogen fixation were much lower in this study. We predicted that microbes may use nitrate and nitrite as the main nitrogen sources for growth rather than nitrogen fixation in the survey area. Allen et al. (2001) found that heterotrophic bacterial nitrate assimilation genes are common and widely distributed in the South Atlantic Bight, the Barents Sea, and the North Pacific Gyre [69]. Martiny et al. (2009) results suggested that, compared with the eastern Pacific Ocean, nitrite and nitrate assimilation genes in uncultured *Prochlorococcus* are higher in the Indian Ocean, where the nitrate concentration is lower [70]. Associated with the numerous occurrences of *Prochlorococcus* in our results, the relative abundance of the nitrate and nitrite reductase genes provided the insight into the role of *Prochlorococcus* in the nitrogen cycles and the feedback loops with carbon flux in the EIO. For nitrogen fixation marker gene *nifH*, a low relative abundance was found in this study. Zehr and Kudela (2011) described that even the dilute populations of N_2_-fixing microorganisms and low N_2_-fixation rates are probably significant relative to in situ nitrogen turnover in oligotrophic open oceans [71]. The present study provided the basic information for understanding the role of nitrogen fixation in the nitrogen cycle.

Furthermore, the second major catalog was determined by genetic information processing based on the KEGG database. The annotated genes is related to the function of replication, recombination, and repair (L) in the COG annotation, indicating the important potential of DNA repair function in the microbial community. A similar phenomenon was revealed in extreme conditions. Xie et al. (2011) detected highly enriched genes for mismatch repair and homologous recombination in the microbial communities from the deep-sea hydrothermal vent environment, suggesting that the microbial communities have evolved extensive DNA repair systems to cope with the extreme environment that subjects genomes to damaging effects by physical or toxic chemical agents [72]. The findings sketch the gene repair potential of the microbial community in the equatorial EIO habitats.

## 5. Conclusions

In this study, we described the genetic diversity and metabolic potential of the microbial communities from the equatorial EIO. Our results confirmed that Cyanobacteria (mainly Oxyphotobacteria) and Proteobacteria contributed most to the microbial community. Among predicted functional categories, we paid close attention to the pathways involved in carbon fixation and the nitrogen cycle. The proportion of these functional genes suggested that the Calvin cycle and assimilatory nitrate and nitrite reduction pathways played crucial roles in energy and resource cycling. This study provides a clue for microbial function potential for biogeochemical cycle in the equatorial EIO. Our future investigation will focus on the contribution and mechanism of microbial communities involving in biogeochemical process, and exploration in the adjustment and evolution of microbial communities to changing environment.

## Figures and Tables

**Table 1:** Temperature (T, °C), salinity (S), chlorophyll *a* (Chl *a*, μg/L) concentration, bottom depth (B. depth, m), and dissolved inorganic nutrients (ammonium, nitrate, nitrite, phosphate; μmol/L) in surface water at sampling stations.

Station	T	S	PO_4_^3−^	NH_4_^+^	NO_2_^−^	NO_3_^−^	SiO_3_^2−^	Chl *a*	B. Depth
E809	29.92	34.48	0.14	0.65	0.07	0.48	1.61	0.23	4656
EQ4	29.62	34.56	0.14	0.28	0.02	0.63	1.82	0.18	4532
EQ10	30.18	34.55	0.097	0.9	0.12	0.66	1.78	0.27	4535

**Table 2:** Metagenome and 16S rDNA and sequencing information and microbial diversity index.

Method	Item	E809	EQ04	EQ10
16S rDNA	Sequences	57,535	54,093	49,114
	No. of OTU	408	362	373
	ACE	431.93	401.85	416.73
	Chao	433.37	398.66	404.60
	Shannon	3.43	2.47	3.20
	Simpson	0.14	0.29	0.12
Metagenome	Clean Bases (G)	16.1	12.6	12.3
	Clean Q20 (%)	97.4	97.5	97.3
	Clean Q30 (%)	92.6	92.8	92.4
	No. of contigs	140,434	176,908	134,477
	No. of predicted gene	533,175	437,671	405,455

**Table 3:** General characteristics of five high-quality reconstructed genomes of microbes.

Accession	Bin	Completeness	Contamination	GC (%)	Lineage	N50	Size (bp)
SAMN18201744	bin.59	99.63	0.543	0.627	Gammaproteobacteria	227,451	3,791,873
SAMN18201743	bin.46	98.8	0.086	0.627	Gammaproteobacteria	109,890	3,789,683
SAMN18201742	bin.15	98.62	0.755	0.586	Gammaproteobacteria	54,890	3,710,402
SAMN18201741	bin.28	98.6	0.836	0.367	Algicola	61,646	3,907,186
SAMN18201740	bin.25	98.24	0.638	0.631	Rhodobacteraceae	39,029	3,209,366

## Data Availability

The data presented in this study are openly available in the NCBI Sequence Read Achieve database (BioProject: PRJNA685401, SUB9213355).

## Supplementary Materials

The following are available online at https://www.mdpi.com/2079-7737/10/3/248/s1, Figure S1: The rarefaction curves of diversity indices were performed. File S1: details of DNA samples measurements.
