# Supplementary material for: The Composition and Primary Metabolic Potential of Microbial Communities Inhabiting the Surface Water in the Equatorial Eastern Indian Ocean
Source: Biology (Basel). 2021 Mar 22;10(3):248. doi: 10.3390/biology10030248 (PMC8005183; doi:10.3390/biology10030248)
Supplement: Supplementary file 1 [file biology-10-00248-s001.zip › Supplementary File S1.docx]

Supplementary details of DNA samples measurements:

1. PCR reactions of 16S rDNA were performed in triplicate 20 μL mixtures containing 4 μL of 5 × FastPfu Buffer, 2 μL of 2.5 mM dNTPs, 0.8 μL of each primer (5 μM), 0.4 μL of FastPfu Polymerase (TransStart, Beijing, China), 0.2 μL of BSA, and 10 ng of template DNA. The thermal profile used for PCR was initial denaturation (95 °C, 2 min), followed by 25 cycles of denaturation (95 °C, 30 s), annealing (55 °C, 30 S), andextension(72°C, 30 S) with a final extension (72 °C, 10 min).
2. PCR products were examined with the 1% agarose gels electrophoresis system. The quality and quantity of extracted DNA were quantified with TBS-380 (Promega, Madison, WI, USA) and NanoDrop2000 (Thermo Scientific, Wilmington, DE, USA), respectively. Amplicons were extracted from 2% agarose gels and purified using the AxyPrep DNA Gel Extraction Kit (Axygen Biosciences, Union City, CA, USA) according to the manufacturer’s instructions and quantified using QuantiFluor™ -ST (Promega, Madison, WI, USA).
3. Raw fastq files of 16 S rDNA were demultiplexed and quality-filtered using QIIME (version 1.17) with the following criteria: (i) The 300 bp reads were truncated at any site receiving an av-erage quality score of <20 over a 50 bp sliding window, discarding the truncated reads that were shorter than 50 bp. (ii) Exact barcode matching, 2 nucleotide mismatches in primer matching, and reads containing ambiguous characters were removed. (iii) Only sequences that overlap longer than 10 bp were assembled according to their overlap se-quence. Reads which could not be assembled were discarded. Operational units (OTUs) were clustered with a 97% similarity cutoff using UPARSE（version 7.1 http://drive5.com/uparse/）, and chimeric sequences were identified and removed using UCHIME.
4. For quality control of metagenomics sequencing, the 3′ and 5′ ends were stripped using SeqPrep (https://github.com/jstjohn/SeqPrep). Low-quality reads (length < 50 bp or with a quality value < 20 or having N bases) were removed by Sickle (https://github.com/najoshi/sickle). De-Bruijn-graph-based assembler SOAPdenovo (http://soap.genomics.org.cn, Version 1.06) was employed to assemble short reads. K-mers, varying from 1/3 to 2/3 of read lengths, were tested for each sample. Scaffolds with a length over 500 bp were retained for statisti-cal tests; we evaluated the quality and quantity of scaffolds generated by each assembly and finally chose the best K-mer which yielded the minimum scaffold number and the maximum value of N50 and N90. Then, scaffolds with a length over 500 bp were extracted and broken into contigs without gaps. Contigs were used for further gene prediction and annotation.
5. Binning analysis is used to assemble the genome sequence of a species according to the frequency of the tetranucleotide and the genome sequence coverage from a large num-ber of metagenomic data. The sequencing depth of each read was calculated.
