# Supplementary material for: The Composition and Primary Metabolic Potential of Microbial Communities Inhabiting the Surface Water in the Equatorial Eastern Indian Ocean
Source: Biology (Basel). 2021 Mar 22;10(3):248. doi: 10.3390/biology10030248 (PMC8005183; doi:10.3390/biology10030248)
Supplement: Supplementary file 1 [file biology-10-00248-s001.zip › Supplementary Figure S1.docx]

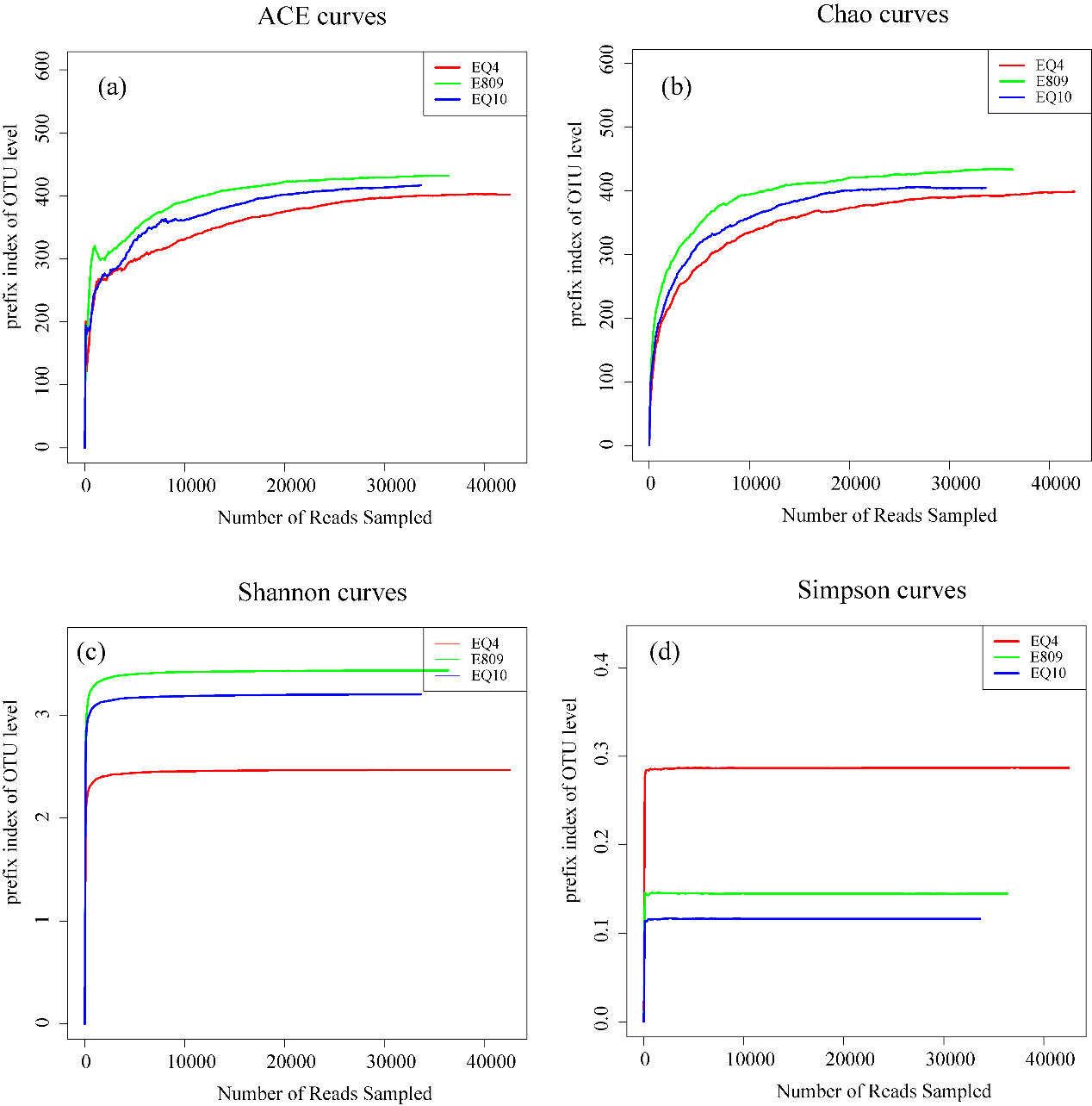


Supplementary Figure S1. The rarefaction curves of diversity indices were performed. (a), rarefaction curve of ACE, (b) rarefaction curve of Chao, (c) rarefaction curve of Shannon, (d) rarefaction curve of Simpson.
